# Synthesis, Characterization, and Antimicrobial and Antiproliferative Effects of CuO-TiO_2_-Chitosan-Escin Nanocomposites on Human Leukemic MOLT4 Cells

**DOI:** 10.3390/nano12213753

**Published:** 2022-10-26

**Authors:** Abozer Y. Elderdery, Abdulaziz H. Alhamidi, Ahmed M. E. Elkhalifa, Maryam M. Althobiti, Nawal Eltayeb Omer, Mahdi H. Alsugoor, Naif Alsuhaymi, Entesar M. Atebien, Siddiqa M. A. Hamza, Badr Alzahrani, Fehaid Alanazi, Suresh Kumar Subbiah, Pooi Ling Mok

**Affiliations:** 1 Department of Clinical Laboratory Sciences, College of Applied Medical Sciences, Jouf University, Sakaka 42421, Saudi Arabia; 2 Health Sciences Research Unit, Jouf University, Sakaka 42421, Saudi Arabia; 3 Clinical Laboratory Sciences Department, College of Applied Medical Science, King Saud University, Riyadh 11451, Saudi Arabia; 4 Department of Public Health, College of Health Sciences, Saudi Electronic University, Riyadh 13316, Saudi Arabia; 5 Department of Clinical Laboratory Science, College of Applied Medical Science, King Saud University, Shaqra 15572, Saudi Arabia; 6 Hereditary Blood Disease Center, Al Ehsaa, Saudi Arabia; 7 Department of Emergency Medical Services, Faculty of Health Sciences, AlQunfudah, Umm Al-Qura University, Makkah 21912, Saudi Arabia; 8 College of Medicine, Department of Pathology, Umm Alqura University Algunfuda, Mecca 24382, Saudi Arabia; 9 Department of Clinical Laboratory Sciences, College of Applied Medical Sciences-AlQurayyat, Jouf University, Sakaka 42421, Saudi Arabia; 10 Centre for Materials Engineering and Regenerative Medicine, Bharath Institute of Higher Education and Research, Chennai 600073, India; 11 Department of Biomedical Science, Faculty of Medicine & Health Sciences, Universiti Putra Malaysia, Serdang 43400, Selangor, Malaysia

**Keywords:** CuO-TiO_2_-chitosan-escin nanocomposites, antioxidant, reactive oxygen species, anticancer, caspase

## Abstract

Nanocomposites comprised of CuO-TiO_2_-chitosan-escin, which has adjustable physicochemical properties, provide a solution for therapeutic selectivity in cancer treatment. By controlling the intrinsic signaling primarily through the mitochondrial signaling pathway, we desired nanocomposites with enhanced anticancer activity by containing CuO-TiO_2_-chitosan-escin. The metal oxides CuO and TiO_2_, the natural polymer chitosan, and a phytochemical compound escin were combined to form CuO-TiO_2_-chitosan-escin nanocomposites. The synthesized nanocomposites were confirmed and characterized using FTIR spectroscopy, TEM, and UV-Vis absorption spectroscopy. A human leukemia cell line (MOLT-4) was used to assess the efficacy and selectivity of nanocomposites. Based on a cytotoxicity study, CuO-TiO_2_-chitosan-escin nanocomposites had inhibition concentrations (IC_50_) of 13.68, 8.9, and 7.14 µg/mL against human T lymphoblast cells after 24, 48, and 72 h of incubation, respectively. Compared with untreated MOLT-4 cells, CuO-TiO_2_-chitosan-escin nanocomposite-treated cells significantly increased (*p* < 0.05) caspase-3, -8, and -9 and decreased the levels of antioxidant enzymes GR, SOD, and GSH. Furthermore, MDA for lipid peroxidase and ROS levels significantly increased (*p* < 0.05) in the treated cells than in the untreated cells. Remarkably, CuO-TiO_2_-chitosan-escin nanocomposite-mediated control of cell cycles were mainly achieved through the activation of caspase-3, -8, and -9.

## 1. Introduction

Lymphoid progenitor cells undergo malignant transformation and proliferate in blood, bone marrow, and extra-medullary sites [1]. Approximately 80% of ALL cases occur in children, whereas adults diagnosed with acute lymphoblastic leukemia (ALL) are in great health danger. Lymphoblastic leukemia, the most common leukemia, develops from precursor T or B lymphoblasts at various phases of development [2].

Immunophenotyping reveals that leukemia-like T-lymphoblast (T-LBLs) contain terminal deoxynucleotidyl transferase (TdT). The lymphoblasts showed small or medium size with sparse cytoplasm, round or twisted nuclei, and small blurry nucleoli [3]. About half of all T-ALL cases in children exhibit recurrent cytogenetic abnormalities; however, these abnormalities are less well defined in T-LBL, and information on typical T-LBL chromosomal abnormalities is limited [4]. The most frequent chromosomal translocations in T-LBL and T-ALL encompass the TCR gene loci on chromosomes 14q11 and 7q34, leading to the fusion of an oncogenic companion gene with the regulatory site of one of the TCR loci and the deregulation of its reciprocal companion gene, such as MYC or RAD51, and TAL1 [5]. FBXW7, NOTCH1, and CDKN2A/CDKN2B mutations on chromosome 9p and 6q defects were identified in the pediatric T-LBL series [6]. Prospective systematic validation is necessary to determine if these molecular markers have consistent prognostic relevance, thereby improving current therapy stratification systems [7].

Chemotherapy has low selectivity and is constrained by dose-limiting effects. Nanomaterials have been used to develop cancer medications, and demonstrate considerable pharmacokinetic and pharmacodynamic benefits in cancer diagnosis and treatment [8]. Nanoparticles enable a more sensitive diagnosis, early detection without extensive tissue removal, long-term therapy monitoring, and removal of cancer cells [9]. CuO nanoparticles are used in biomedical applications for gene and drug delivery, and as a catalyst support [10]. Nonetheless, an investigation of TiO_2_ nanoparticles suggested that glutathione levels may be involved in oxidative DNA damage. This damage led to increased lipid peroxidation and reactive oxygen species (ROS) formation [11]. Various cultured cells have been shown to die after exposure to ZnO NPs and CuO NPs, which produce ROS [12].

TiO_2_ NPs and ZnO/CuO NPs are the most efficient metal oxide semiconductor catalysts. TiO_2_ NPs and ZnO/CuO NPs exhibit a limited light response because of their large bandgaps and significant electron and hole recombination [13]. Several attempts have been made to combine TiO_2_ and CuO/ZnO catalysts with narrow bandgap metallic oxides intrinsically irradiated by visible light. To improve their performance, researchers have developed a visible light catalyst [14]. Metal oxides, such as CuO, have a low bandgap (1.208 eV) and are excellent gas-phase catalysts [15]. Studies have demonstrated that the absence of H_2_O_2_ reduced the catalytic activity of CuO. Despite its wide range of applications, ZnO combined with CuO has limited photodegradation properties [16].

Hydrothermal processing was used to produce highly effective and low-cost photocatalysts. During this procedure, two CuO molecules and one TiO_2_ molecule are bonded to form a heterojunction CuO-TiO_2_ nanocomposite [17]. Electrons are extracted from TiO_2_ using CuO, suppressing e/h+ recombination, and then transferred to split water, leading to increased hydrogen production. Through an unique chemical vapor deposition (CVD) technique based on the concurrent growth of CuO matrices in O_2_ atmospheres and TiO_2_ overdispersion in H_2_O atmospheres, we created the CuO-TiO_2_ nanocomposites on Al_2_O_3_ substrates [18].

Chitosan is nontoxic, biocompatible, biodegradable, and shows anti-bacterial property. This organic polymer is the second most common biopolymer on the planet. As a biosorbent, chelating agent, and support for other nanomaterials, chitosan’s wide range of applications have been intensively researched [19]. Chitin is typically recovered from crab waste, squid, and oysters; chitosan, a polycationic compound with antibacterial properties, is used for protective finishes [20]. Chitin deacetylates to chitosan by creating a linear polysaccharide that is made up of sporadic distributions of -(1-4)-linked d-glucosamine (deacetylated) and N-acetyl-d-glucosamine (acetylated). Chitosan has been extensively used in tissue engineering because it is biocompatible and biodegradable [19]. TiO_2_ has been shown to improve the mechanical, physical, and biological properties of nanocomposites containing chitosan because it is biocompatible with an array of organic and inorganic compounds [21].

Escin, an active component, is found in Horse chestnut and *Aesculus hippocastanum*. It has been used as a traditional medicine for centuries to treat hemorrhoids, varicose veins, hematomas, and venous congestion [22]. Escin, discovered in various forms in 1953, has anti-inflammatory, anti-edematous, and venotonic effects. Additionally, it has demonstrated efficacy as a complement or substitute for compression therapy in the treatment of endothelial hypoxia [23]. Early studies have reported that the metallic particles CuO and TiO_2_ have anti-cancer properties. Anti-oxidant properties are exhibited in natural polymers such as chitosan and phytocompounds such as Escin. In this study, however, we aimed to synthesize novel nanocomposites using these four materials, i.e., CuO, TiO_2_, chitosan, and escin, and investigate the anti-cancer properties of these nanocomposites.

According to our literature search, the effect of biosynthesized CuO-TiO_2_-chitosan-escin nanocomposites on cancer cells has never been studied. CuO-TiO_2_-chitosan-escin nanocomposites were produced, and the materials were evaluated using several approaches such as XRD, FTIR, and UV spectrum, as well as SEM and TEM images. The cytotoxicity of nanocomposites against MOLT-4 cells was examined. In MOLT-4 cells, we also assessed the caspase proteins. The mechanism of anti-cancer efficacy of biosynthesized CuO-TiO_2_-chitosan-escin nanocomposites against human T lymphoblast (MOLT-4) intrinsic signaling was explored, with a focus on the mitochondrial signaling pathway. Remarkably, the functional response was significantly improved after the inclusion of CuO-TiO_2_-chitosan-escin nanocomposites, highlighting the technological applications of the current CuO–TiO_2_–chitosan nanosystems as appealing possibilities.

## 2. Materials and Methods

### 2.1. Chemicals

The chemicals utilized in this work were obtained from Sigma-Aldrich, St. Louis, MO, USA: Cu(NO_3_)_2_·6H_2_O (99–104% purity), TiO_2_ ≥99.5% purity, Chitosan (deacetylated) ≥75% purity, and Escin powder ≥95% purity.

### 2.2. Synthesis of Copper Oxide-Titanium Dioxide-Chitosan-Escin Nanocomposites

First step solution: in a 250 mL beaker, 50 mL of TiO_2_ NPs (500 mg) were mixed with 50 mL of Cu(NO_3_)_2_.6H_2_O (0.1 M), and 50 mL of a 1% acetic acid aqueous solution mixture was used to dissolve the 500 mg of chitosan. We obtained a homogenous CuO-TiO_2_-chitosan mixed solution. Second step solution: To perform the first step, we mixed the solution (CuO-TiO_2_-chitosan) with 50 mL of escin (50 mg). We then added 0.1 M of sodium hydroxide (50 mL) solution drop-by-drop into the CuO-TiO_2_-chitosan-escin mixture. We obtained brown precipitate, and a magnetic stirrer was used to agitate the precipitate at room temperature for three hours. The resultant nanopowder was thoroughly cleaned using ethanol and deionized water. The precipitate were centrifuged at −3 °C for 40 min at 15,000 rpm, and finally calcined at 600 °C for 5 h [24].

### 2.3. Characterization Analysis

XRD (model: X’PERT PROPANalytical, Cambridge, MA, USA) were utilized to characterize the obtained CuO-TiO_2_-chitosan-escin sample. Using a monochromatic CuK diffraction beam of wavelength 1.5406, the diffraction patterns for the CuO-TiO_2_-chitosan-escin sample were observed in two ranges between 25° and 80°. The CuO-TiO_2_-chitosan-escin nanocomposites were analyzed using a FE-SEM (Carl Zeiss Ultra 55 FESEM, Oberkochen, Germany) with EDAX spectrometry (model: Inca, High Wycombe, UK). The CuO-TiO_2_-chitosan-escin morphologies were studied with a TEM (Tecnai F20 model, Columbus, OH, USA) apparatus operating at an accelerating voltage of 200 kV. The FTIR spectrum was acquired using a Perkin-Elmer spectrometer (Waltham, MA, USA) in the wavenumber range of 400–4000 cm^−1^. The Lambda 35 spectrometer (Waltham, MA, USA) was used to investigate the absorption spectra of CuO-TiO_2_-chitosan-escin nanocomposites in the 200–1100 nm region. A Perkin Elmer-LS 14 spectrometer (Waltham, MA, USA) was used to capture the photoluminescence (PL) spectra [24].

### 2.4. Anti-Bacterial Activity

Using the well diffusion method, Gram-positive (*S. pneumoniae* and *S. aureus*) and Gram-negative (*E. coli* and *K. pneumonia*) bacterial strains were tested for the anti-bacterial activity of nanocomposites. The constructed Petri plate was rinsed with 25 mL of agar media. Separate cultures of bacterial pathogens were grown on sterile Mueller Hinton agar media. CuO-TiO_2_-chitosan-escin nanocomposites at 1, 1.5, and 2 mg/mL concentrations were dispersed in a 5% sterilized dimethyl sulphoxide solution. We also incubated the testing Petri plates overnight at 37 °C and evaluated the inhibition zones after 24 h. Amoxicillin (30 µg/mL), a common antibiotic, served as a positive control, and triplicate assays were performed [25].

### 2.5. Anti-Fungal Properties

The anti-fungal activity was evaluated using the agar well diffusion technique and potato dextrose agar (PDA). The *C. albicans* strain was cultured onto a PDA agar plate by streaking 2–3 times and rotating the plate at 60° for each streak to ensure uniform inoculum distribution. On the inoculation plates, test CuO-TiO_2_-chitosan-escin nanocomposites at doses of 1, 1.5, and 2 mg/mL were placed in wells and sustained at 30 °C for 24 h under visible light. The inhibitory zone were detected using amphotericin B as a positive control. Three independent assays were conducted [26].

### 2.6. Cell Culture

Human ALL cells (MOLT-4) were cultured in RPMI 1640 medium comprising 10% FBS, 100 IU/mL penicillin, and 100 g/mL streptomycin. The cells were sustained at 37 °C in a CO_2_ environment with 5% humidity.

### 2.7. Cell Viability Assay

Utilizing MTT as an indicator chemical, CuO-TiO_2_-chitosan-escin nanocomposites were assessed for their cytotoxic effect on the cells. During a 24-h incubation period, MOLT-4 cells were grown on 96-well plates. The cells were administered diverse doses of nanocomposites for 24, 48, and 72 h. The cells were then cleansed with PBS solution three times. The cells were sustained in a shady place at 37 °C for 4 h with MTT solution (0.5 mg/mL). The formazone crystals were dissolved using dimethyl sulfoxide (DMSO), and the absorbance at 595 nm was detected using a UV spectrophotometer (Thermo Fisher Scientific, Waltham, MA, USA). Based on the total number of viable cells, the viability of cells was calculated [27].

### 2.8. Assessment of the Intracellular Activity of GR, SOD, GSH, and MDA

The glutathione reductase (GR) activity of MOLT-4 cells incubated with CuO-TiO_2_-chitosan-escin nanocomposites was used to calculate oxidative stress. MOLT-4 cells were loaded on 6-well plates at 2 × 10^6^ cells/well and administered with the IC_50_ (7 and 14 µg/mL) of nanocomposites for 24 h. After harvesting, the cells were washed with PBS. The GR content in the cells was determined using Abcam kits and absorbance measurements at 412 nm. Similarly, for other enzymes, MOLT-4 cells were administered for 24 h to the IC_50_ of nanocomposites (7 and 14 µg/mL). Following treatment, the cells were extracted. The cells were washed in PBS, then lysed in lysis buffer before being centrifuged at 12,000 rpm for 10 min at 4 °C and collecting the supernatant. We used manufacturer-specified assay kits to measure the intracellular SOD, and the concentrations of SOD in cell lysates were detected by the BCA approach. The activity of SOD was determined using hypoxanthine and xanthine oxidase systems [28]. A unit of SOD activity is calculated as U/mg protein and represents the quantity of enzyme needed to stop 50% oxidation in a reaction solution. The rate constant of H_2_O_2_ decomposition was used to calculate catalase activity as U/mg protein [29]. Lipid peroxidation and MDA status were detected and expressed in nmol/mg protein by using a lipid peroxidation (MDA) assay kit (ab118970, Abcam; Waltham, MA, USA) (Colorimetric/Fluorometric) as per the guidelines of manufacturer. The intracellular GSH content in whole cells was detected using an enzymatic recycling approach involving GR and 5-dithio-5′,5′-dinitrobenzoic acid (DTNB), in which DTNB oxidized GSH and NADPH, and GSH was expressed as nmol/mg protein [30].

### 2.9. Intracellular ROS Estimation

A population of 5 × 10^3^ MOLT-4 cells/well was seeded into 96-well plates, and the cells were grown overnight. A cellular ROS detection assay kit (Abcam, Waltham, MA, USA) with 10 µM 2.7-dichlorofluorescein diacetate was used to stain the cells for 45 min by using nanocomposites (7 and 14 µg/mL) for 24 h.

### 2.10. Morphological Assessment of Apoptotic Cells

The viable and deceased control cells and CuO-TiO_2_-chitosan-escin nanocomposite-treated cells were assessed using a dual staining approach. AO/ PI dye stains both viable and dead cells, whereas PI dye only cells with impaired membrane structure. The MOLT-4 cells were cultivated in 6-well plates and incubated for 24 h with two dosages of nanocomposites (7 and 14 µg/mL). After that, the treated cells were rinsed with PBS and loaded with AO (100 mg/mL) and PI (100 mg/mL) dyes for 3 min at room temperature. Following the incubation period, the treated cells were rinsed with PBS then viewed and recorded using ZOE Fluorescent Cell Imager with 20× magnification (BioRad, Hercules, CA, USA) [31].

The DAPI staining was executed as per the guidelines of manufacturer, with a few modifications. The MOLT-4 cells (2 × 10^6^ cells/well) were maintained overnight in 6-well plates in a serum-free medium with CuO-TiO_2_-chitosan-escin nanocomposites (at IC_50_ concentrations of 7 and 14 µg/mL). Following incubation, the cells were fixed with 4% paraformaldehyde, permeabilized with 0.1% Triton X-100 in PBS, and the nuclei were stained for 20 min in the dark with 10 L DAPI in PBS (2.5 g/mL). Morphological changes were visualized using the ZOE Fluorescent Cell Imager (BioRad, Hercules, CA, USA) with 20× magnification [32].

Similarly, following the aforementioned treatments, the cells were fixed with 4% paraformaldehyde in PBS for 20 min and permeabilized with 0.5% Triton X-100 for 15 min. The cells were treated with 10 µL PI to identify necrotic cells. The cells were then examined and photographed. PI was immediately detected using the ZOE Fluorescent Cell Imager (BioRad, Hercules, CA, USA) with 20× magnification [33].

### 2.11. Apoptosis and Viability of Cells Detected by FACS Using Annexin-V FITC/PI

A density of 2 × 10^5^ cell/well was seeded into 24-well plates. The IC_50_ concentration (7 and 14 µg/mL) was administered for 24 h to the cells to explore the potential apoptosis caused by CuO-TiO_2_-chitosan-escin nanocomposites. Using BD FACSCanto II, (BD Bioscience, Franklin Lakes, NJ, USA) and BD FACSDiva software v. 6.1.3, (BD Bioscience, Franklin Lakes, NJ, USA) we analyzed apoptotic cells by Annexin V staining using the Annexin V-FITC apoptosis detection kit (BD Bioscience) as per the manufacturer’s instructions. For each sample, 10,000 cells in total were examined. In the bottom right quadrant, only Annexin V-FITC-positive cells are present, indicating early apoptosis. Late apoptotic cells labeled with Annexin V-FITC and propidium iodide (PI) are visible in the top right quadrant. In the top left quadrant, necrotic cells that are only positive for PI staining are present.

### 2.12. Caspase-3, -8, and -9 Activity Assay

Caspase activities were measured using chromogenic tests with caspase-3, -8, and -9 kits, as directed by the manufacturer (Calbiochem, Merck, Kenilworth, NJ, USA). We created cell lysates by incubating 2 × 10^6^ cells/well for 10 min on ice in the cell lysis solution following treatment with different doses of CuO-TiO_2_-chitosan-escin nanocomposites (7 and 14 µg/mL) and DOX (5 µM). Using Lowry’s method with BSA as a standard, the protein content in the supernatants were detected by centrifuging the lysate for 1 min at 10,000× *g*. Each test was conducted with diluted cellular protein in 50 µL cell lysis buffer. The cellular protein was then treated for 2 h at 37 °C with 5 µL of 4 mM pNA substrate DEVD-pNA (caspase-3 activity), IETD-pNA (caspase-8 activity), and LEHD-pNA (caspase-9 activity). Caspase activity was determined by the cleavage of the aforementioned substrates to free pNA. The absorbance of cleaved substrates at 405 nm were detected using a microtiter plate reader. In this study, we calculated the relative caspase activity using the following formula: Activity = OD × d εmM × t × v, where εmM is 10.5, v signifies the sample volume in mL, d signifies the dilution factor, and t signifies the reaction time in min [34].

### 2.13. Statistical Analysis

All outcomes were examined using GraphPad Prism version 5, San Diego, CA, USA and depicted as mean ± SD. With SPSS software, we calculated variations between the two groups using the student’s t-test, and significance was tested at * *p* < 0.05, ** *p* < 0.01, and *** *p* < 0.001.

## 3. Results

### 3.1. Characterization of CuO-TiO_2_-Chitosan-Escin Nanocomposite

**Figure 1:**
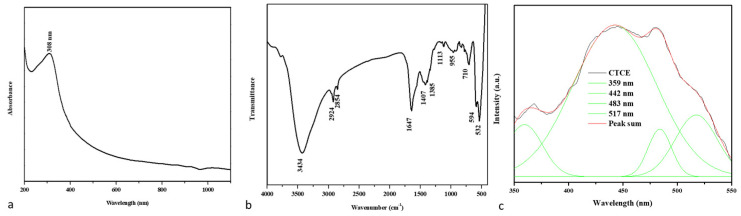
An analysis of the spectra of CuO-TiO_2_-chitosan-escin nanocomposites. The UV-vis spectrum of nanocomposites (**a**). FTIR transmittance vs. wavenumber plot of nanocomposites (**b**). Photoluminescence spectra from nanocomposites (**c**).

Figure 1a shows the UV-visible absorbance spectrum of the developed nanocomposites. The absorption edge detection peak was observed at 308 nm, indicating the presence of an optical bandgap in CuO-TiO_2_-chitosan-escin nanocomposites. In the literature, the CuO absorption peak at 305 nm is most likely accredited to CuO semiconductors’ SPR excitation [35].

Figure 1b shows the FTIR spectrum of nanocomposites produced. The broad -OH and -NH peaks with hydrogen bonds were identified in the chitosan peaks at 3434 and 1647 cm^−1^, confirming the presence of the amide I group (C-O stretching along with the N-H deformation mode). The COO- group of the carboxylic acid salt could be accountable for the peak at 1385 cm^−1^ [36]. As can be seen in the graph below, escin produced distinct peaks caused by its C-H group: asymmetrical peaks at 2924 cm^−1^ and symmetrical peaks at 2854 cm^−1^, respectively; a stretching peak at 1113 cm^−1^; and an out-of-plane bending peak at 955 cm^−1^ [37,38]. The stretching vibration of the Cu-O and Ti-O bonds also appeared at nearly 710, 594, and 532 cm^−1^ [39]. The FTIR spectrum revealed that CuO-TiO_2_ strongly interacted with chitosan and escin molecules because of the electrostatic interaction among the CuO-TiO_2_-chitosan-escin nanocomposites.

The PL spectrum revealed the efficiency of charge carrier entrapment, migration, transfer, and separation. It may be lifetime electron/hole pair recombination in semiconductor materials. A PL spectrum of the nanocomposites with a 325-nm excited wavelength is shown in Figure 1. These nanocomposites have PL emission values of 359, 442, 483, and 517 nm. The UV emission at 359 nm could be attributed to electron recombination in the conduction band and hole recombination in the valence band. The visible emission maxima at 442, 483, and 517 nm could be caused by band edge-free excitons [40]. The excitonic PL signal in the 400–550 nm region in photocatalysts is associated with surface oxygen vacancies or defects [41].

**Figure 2:**
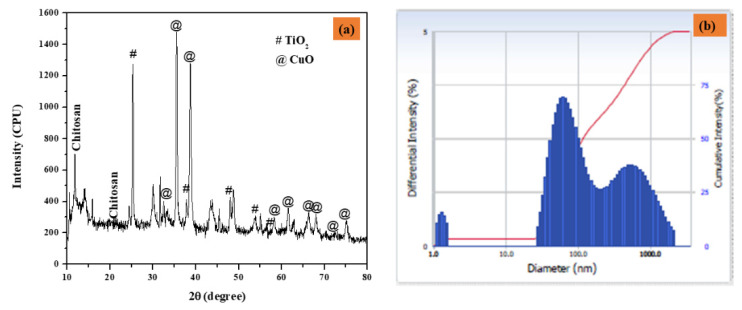
The XRD pattern of nanocomposites (**a**) and particle size distribution as a function of particle number (**b**) were calculated using DLS.

Figure 2a depicts the XRD patterns of CuO-TiO_2_-chitosan-escin nanocomposites. The diffraction pattern of the monoclinic crystal structure of CuO phase (JCPDS 05-066) peaks at 2θ values of 32.43°, 35.72°, 38.82°, 48.80°, 58.38°, 61.50°, 65.93°, 66.35°, 68.002°, 72.46°, and 75.178° are connected to (-110), (002), (111), (-202), (020), (202), (113), (-311), (-113), (311) and (004), respectively. Furthermore, the TiO_2_ peaks detected at 25.36°, 47.94°, 53.95°, and 56.66° are connected to (101), (004), (105), and (200) hkl planes (JCPDS 21-1272), indicating that TiO_2_ is in the anatase phase. The non-crystalline chitosan peaked at 10.49° and 20.98°. Furthermore, the escin diffraction peaks were found at 15.83°. According to these findings, the formation of the CuO-TiO_2_-chitosan-escin phase is ascribed to both steric effects and intermolecular hydrogen bonding among the CuO-TiO_2_-chitosan-escin matrixes. According to the Debye-Scherrer formula, the average crystallite size of the CuO-TiO_2_-chitosan-escin was 45 nm [42].

The dynamic light scattering (DLS) nano plus instruments were used to determine the average size of the nanocomposites. Under stable conditions, the Z-average mean diameter (d [nm]) of the CuO-TiO_2_-chitosan-escin nanocomposites was 116 nm, and the polydispersity index (PDI) was 0.283 with a refractive index of 1.3328. The peak, however, strongly indicates that the particles were mono-dispersed, as seen in Figure 2b.

**Figure 3:**
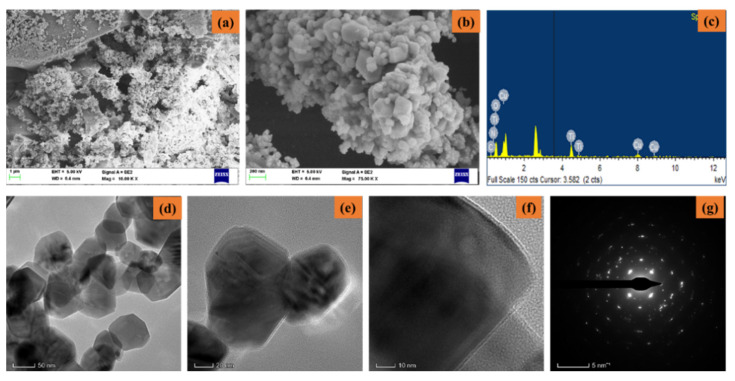
Electron microscopic pattern of nanocomposites: Lower and higher magnifications of FESEM (**a**,**b**), EDAX spectra (**c**), TEM images (**d**–**f**), and SAED pattern (**g**) of nanocomposites.

Figure 3a–g shows the FESEM/EDAX/TEM/SAED pattern of synthesized CuO-TiO_2_-chitosan-escin nanocomposites. From the lower and higher magnification FESEM images (Figure 3a,b), the synthesized CuO-TiO_2_-chitosan-escin nanocomposites formed into octagonal nanostructures with an average size of nanoparticles of 50–60 nm. The EDAX spectrum (Figure 3c) of the CuO-TiO_2_-chitosan-escin nanocomposites had an atomic percentage observed at 14.89% (C), 8.65% (N), 10.80% (Cu), 15.00% (Ti), and 50.65% (O). The TEM images (Figure 3d–f) of CuO-TiO_2_-chitosan-escin nanocomposites revealed octagonal-like nanostructures. However, chitosan and phytocomponent escin were coated in a layer-like manner on the CuO-TiO_2_ nanoparticles, as seen in the TEM images (Figure 3f). The CuO-TiO_2_-chitosan-escin nanocomposite interactions were attributed to the strong intermolecular hydrogen bonds and electrostatic interaction within the chitosan and escin molecules, which were the surface charges of CuO-TiO_2_ nanoparticles. Figure 3g shows the selected area of the SAED pattern of the prepared CuO-TiO_2_-chitosan-escin nanocomposites (Figure 3g).

### 3.2. Nanocomposites of Copper, TiO_2_, Chitosan, and Escin Exhibit Anti-Microbial Properties

**Figure 4:**
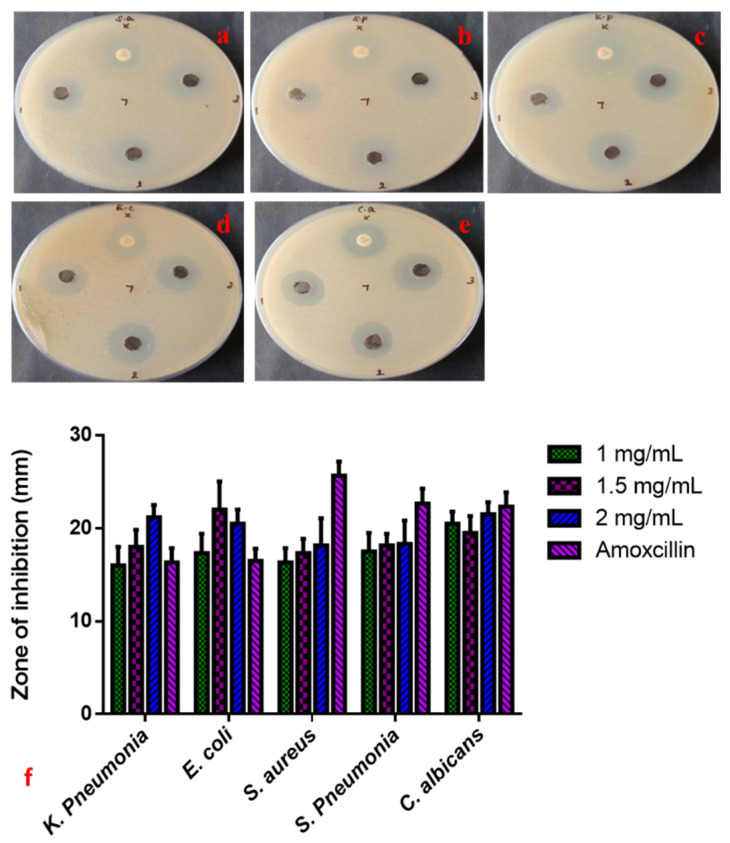
Anti-microbial activity of CuO-TiO_2_-chitosan-escin nanocomposites. Anti-bacterial activity (**a**–**e**) and zone of inhibition (**f**) of CuO-TiO_2_-chitosan-escin nanocomposites treated with *S. aureus* (**a**), *S. pneumonia* (**b**), *K. pneumonia* (**c**), *E. coli* (**d**), and *C. albicans* (**e**) strains.

The anti-microbial properties of CuO-TiO_2_-chitosan-escin nanocomposites were evaluated using a simple well diffusion method. We also investigated the anti-microbial efficacy of various concentrations of CuO-TiO_2_-chitosan-escin nanocomposites (1, 1.5, and 2 mg/mL) against *S. aureus, S. pneumonia, K. pneumonia, E. coli,* and *C. albicans* strains, as shown in Figure 4a–e. Both the CuO-TiO_2_-chitosan-escin nanocomposites and the conventional antibiotic amoxicillin show anti-microbial effects. Increasing the concentration of CuO-TiO_2_-chitosan-escin nanocomposites also increased the anti-microbial effects (Figure 4). The anti-microbial mechanisms for the intercellular activity of CuO-TiO_2_-chitosan-escin nanocomposite are explained as follows. CuO-TiO_2_-chitosan-escin nanocomposites could bind to the surface of microbial membranes, inhibiting protein synthesis and nutrient uptake and, consequently, reducing cell growth and viability. Moreover, the electrostatic communication of CuO-TiO_2_-chitosan-escin nanocomposites on the bacterial surface membrane might have a positive, negative, or neutral charge, which could lead to the collapse of the electrostatic balance on the bacterial surface and create a hole pair on the outer membrane, subsequent in the protein leakage, DNA, RNA, and lipids and, ultimately, cell death. Another possible bactericidal mechanism of nanocomposites could be due to the generation of ROS, which interacted with bacterial membranes, ensuing in the lysis of cell walls and, ultimately, cell death [43].

### 3.3. Growth Inhibition and Viability

**Figure 5:**
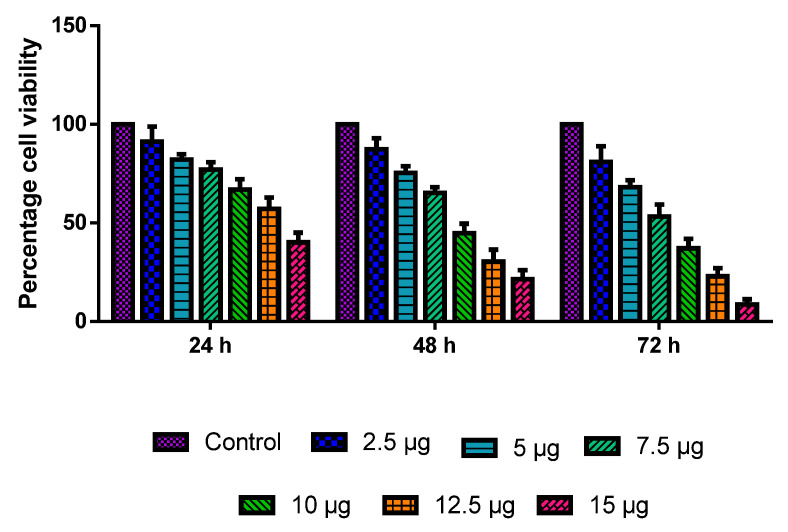
MOLT-4 cells are cytotoxic to nanocomposites of CuO-TiO_2_-chitosan-escin. A range of doses (2.5, 5, 7.5, 10, 12.5, and 15 µg/mL) of CuO-TiO_2_-chitosan-escin nanomaterials were used for 24 h, 48 h, and 72 h in order to evaluate their effects on MOLT-4 cells. The MTT assay was performed on the cells, and the outcomes are illustrated as the mean ± SD of triplicates.

The MOLT-4 cells were administered with various dosages of CuO-TiO_2_-chitosan-escin nanocomposites for 24, 48, and 72 h. The CuO-TiO_2_-chitosan-escin nanocomposites induced dose- and time-dependent cell death (Figure 5) with IC_50_ values of 13.68, 8.9, 7.14 µg/mL in 24, 48, and 72 h, respectively. The outcome demonstrated a substantial reduction in cell growth and viability at a high dose after 72 h.

### 3.4. Biochemical Assay of Oxidative Stress

**Figure 6:**
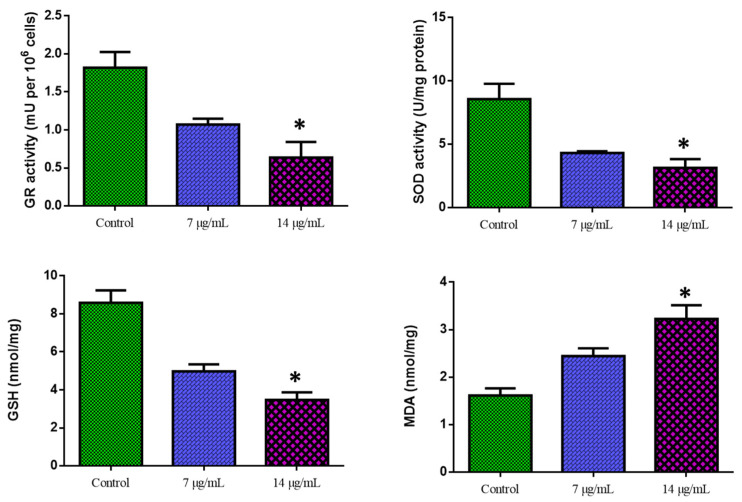
Cuo-TiO_2_-chitosan-escin nanocomposite-induced antioxidant responses in MOLT-4 cells. Using colorimetric analysis, we determined the active levels of GR, SOD, GSH, and MDA in MOLT-4 cell lines after treatment with Cuo-TiO_2_-chitosan-escin nanocomposites for 24 h. The data represented the activities of GR, SOD, GSH, and MDA enzymes and were illustrated as the mean ± SD of triplicates. *n* = 3, * *p* < 0.05 compared with control cells.

**Figure 7:**
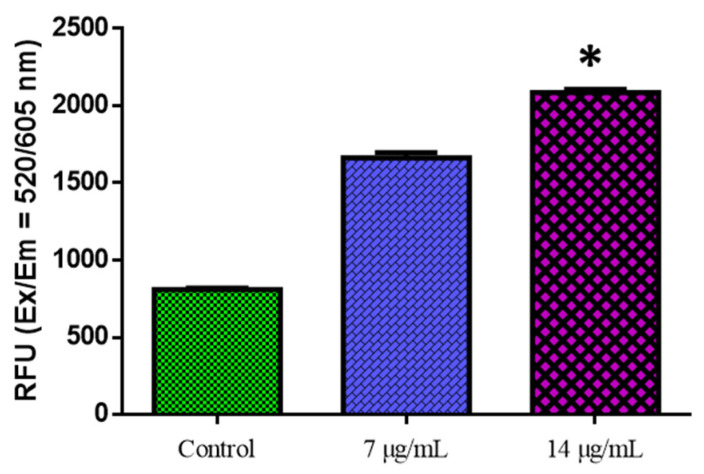
The detection of reactive oxygen species in MOLT-4 cells. MOLT-4 cells were administered for 24 h with or without the IC_50_ dose of nanocomposites. The fluorescence signal was measured using the SpectraMax M2 Series Multi-Mode Microplate Reader in bottom read mode at Ex/Em = 520/605 nm (cut off 590 nm) (Molecular Devices). * *p* < 0.05 compared with control cells.

Figure 6 shows the outcomes of the biochemical experiment on oxidative stress. Compared with the control cells, CuO-TiO_2_-chitosan-escin nanocomposites significantly suppressed GR activity in a dose-dependent manner, significantly inhibited SOD and GSH activities, and considerably reduced MDA levels. This reduction in MDA levels indicates lipid peroxidation inhibition (Figure 6). ROS production was detected using DCFH-DA in MOLT-4 cells. Green fluorescence was the strongest in CuO-TiO_2_-chitosan-escin nanocomposites, suggesting that the nanocomposites could cause oxidative stress in tumor cells by boosting ROS production through NADPH oxidation (Figure 7).

Oxidative stress is typically caused by increased ROS production or decreased ROS clearance, leading to oxidative injury to macromolecules and cells. Though most natural compounds are antioxidants, these compounds can act as pro-oxidants in the tumor cell microenvironment. These chemicals, like resveratrol, are regarded as anticancer pro-oxidant natural products. The potential of CuO-TiO_2_-chitosan-escin nanocomposites to trigger oxidative stress and boost ROS generation in MOLT-4 cells strengthens our findings (Figure 7).

### 3.5. Confirmation of Apoptosis by Morphological Changes

**Figure 8:**
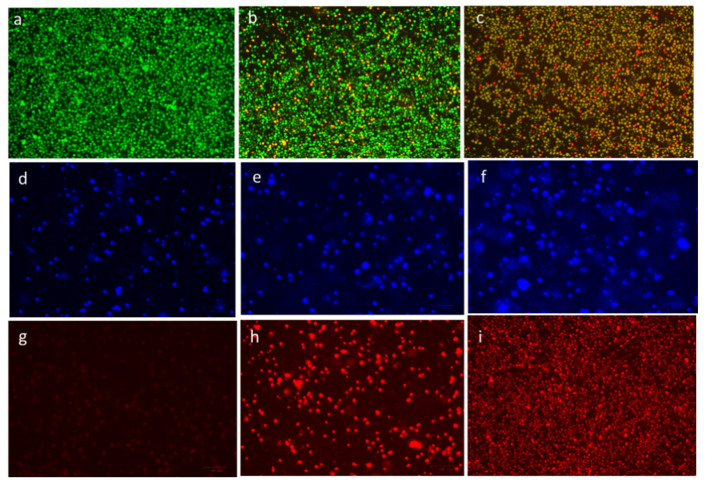
Detection of apoptosis through fluorescence microscopy in cells after treatment with CuO-TiO_2_-chitosan-escin nanocomposites. In vitro treatment with nanocomposites (7 and 14 µg/mL) was carried out for 24 h in MOLT-4 cells. AO/PI was used to stain the cells on the top panels; DAPI was used for the middle panel, and propidium iodide (PI) was used for the lower panel. In the panels on the right, untreated cells served as controls (**a**,**d**,**g**), and the middle panels (**b**,**e**,**h**) were treated with Cuo-TiO_2_-chitosan-escin nanocomposites (7 μg/mL). Left panel samples (**c**,**f**,**i**) were treated with Cuo-TiO_2_-chitosan-escin nanocomposites (14 μg/mL). These images were obtained at 20× magnification; the experiments were conducted in triplicates; the scale bar was set to 100 μm.

The ability of CuO-TiO_2_-chitosan-escin nanocomposites to induce apoptosis in MOLT-4 cells was confirmed using fluorescence microscopy. MOLT-4 cells exhibited morphological changes after 24 h of treatment with CuO-TiO_2_-chitosan-escin nanocomposites (Figure 8). In the control cells, no significant apoptosis was observed, as evidenced in Figure 8a. AO nuclear staining of crescent-shaped or granular cells indicated early stage apoptosis, as evidenced in Figure 8b, with yellow-green AO nuclear staining located asymmetrically within the cells. When the nanocomposite concentration was also increased during the treatment, early stage of apoptosis was increased in cells. Late-stage apoptotic cells were observed with concentrated and asymmetrically localized orange nuclei. As shown in Figure 8c, PI staining revealed the disintegration of necrotic cells as the cells demonstrated an increase in volume and uneven red fluorescence around their peripheries.

The chromatin condensation that takes place during apoptosis can be used to identify apoptotic cells [44,45]. When stained with DAPI, condensed chromatin in nuclei increases the maximum intensity of DAPI pixels. Chromatin condensation also diminishes the size of the nucleus and, thus, the nucleus area. The cells treated with CuO-TiO_2_-chitosan-escin nanocomposites had higher DAPI levels and lower nuclear areas (Figure 8e,f). CuO-TiO_2_-chitosan-escin nanocomposites, on the other hand, interacted with cells with condensed chromatin (Figure 8d). After 24 h of incubation, we observed an elevation in the cell numbers with condensed nuclei, indicating an elevation in the apoptotic cell numbers (Figure 8f). Apart from this, DAPI staining demonstrated that all treated MOLT-4 cells gradually lost their shape. In contrast, the control cells remained undamaged and uniform in shape. Most cells remained intact until 24 h of incubation but lost shape after 24 h. DAPI staining revealed apoptotic morphological changes in cells treated with CuO-TiO_2_-chitosan-escin nanocomposites.

Because of the inability of PI to pass through living cells, PI fluorescence can only be detected in dead cells [46]. Few PI-positive MOLT-4 cells were present in the absence of the CuO-TiO_2_-chitosan-escin nanocomposites (Figure 8g). The CuO-TiO_2_-chitosan-escin nanocomposites treatment, however, significantly increased MOLT-4 cell death when compared with the control cells (Figure 8h,i). The CuO-TiO_2_-chitosan-escin nanocomposites reduced the number of necrotic dead cells, indicating that they were cytotoxic and killed cells.

### 3.6. Nanocomposites of CuO-TiO_2_-Chitosan-Escin Induce Apoptosis in Cells

**Figure 9:**
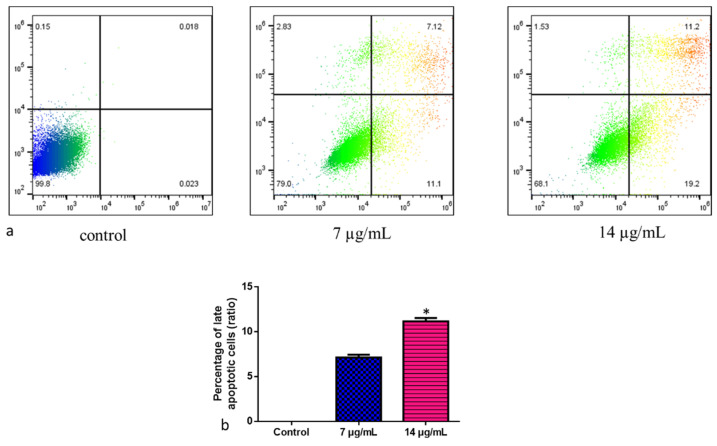
Representative dot plots and histograms of MOLT-4 cells stained with Annexin VFITC and PI. (**a**) The cell lines MOLT-4 were treated or left untreated (control) with nanocomposites (7 and 14 µg/mL) for 24 h. Apoptosis was then evaluated by FACS study. The percentage of viable cells were reported in Q4; the amount of early apoptotic cells were reported in Q3; the amount of late apoptotic cells was reported in Q2; the percentage of non-viable necrotic cells was reported in Q1. A minimum of 3 separate experiments were carried out in triplicate for each sample. 2 × 10^4^ events were acquired for each sample. (**b**) Values are expressed as mean ± SD. * *p* < 0.05 vs. control.

The cell deaths revealed that CuO-TiO_2_-chitosan-escin nanocomposites caused either apoptosis or necrosis. This study used annexin V/PI double-labeling and flow cytometry to analyze phosphorylated serine externalization. To detect apoptotic cells, we used distinct double staining patterns: viable cells (Annexin V- and PI-, lower left square), early apoptotic cells (Annexin V+ and PI-, lower right square), late apoptotic cells (Annexin V+ and PI+, upper right square), and necrotic cells (Annexin V- and PI+, upper left square). The addition of the nanoparticles (Figure 9a) increased the amount of late apoptotic cells. Approximately 7.12% of apoptotic cells were induced by CuO-TiO_2_-chitosan-escin nanocomposites at a dose of 7 µg/mL, whereas 11.2% were induced at a dose of 14 µg/mL (Figure 9b). The control cells had 0.018% late apoptotic cells. CuO-TiO_2_-chitosan-escin nanocomposites inhibited MOLT-4 cell proliferation and induced apoptosis, consistent with MTT results.

### 3.7. Apoptosis Mediated by a Caspase-Dependent Pathway

**Figure 10:**
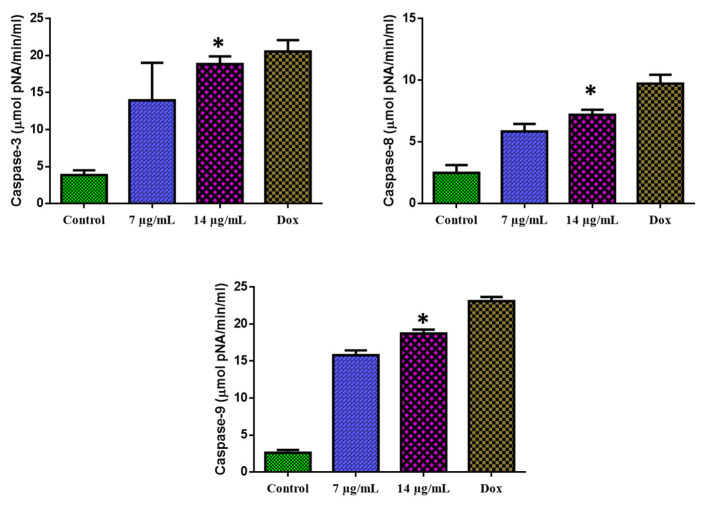
Activation of caspase-3, -8, and -9 in MOLT-4 cells. The level of pro-apoptotic proteins (Caspase-3, -8, and -9) expressed in MOLT-4 cells following treatment with the IC_50_ concentration (7 and 14 µg/mL) of nanocomposites and untreated control cells were measured. Values were illustrated as the mean ± SD of triplicates. * *p* < 0.05 vs. control.

The 24-h exposure of MOLT-4 cells with CuO-TiO_2_-chitosan-escin nanocomposites substantially augmented the caspase-3, -8, and -9 functions (Figure 10). MOLT-4 cells were found to express caspase-3, -8, and -9 enzyme activities. We observed that with the increasing concentration of CuO-TiO_2_-chitosan-escin nanocomposites, caspase-3, -8, and -9 enzyme activity also increased.

## 4. Discussion

In recent decades, anti-cancer agents with fewer side effects and higher efficacy have become increasingly sought. The role of natural plant-based agents in discovering their potential in cancer phytotherapy has always been in the spotlight [47]. Bimetallic nanocomposites using plant-derived phytochemicals have recently gained much attention. Compared with untreated cells, a combination of plant-derived phytochemicals and metal oxides in the form of nanocomposites is more effective against cancer and other clinical applications [48]. CuO-TiO_2_-chitosan-escin nanocomposites were synthesized and characterized in this study. Testing the nanocomposites against the MOLT4 blood cancer cell line experimental model revealed that these nanocomposites possess anti-microbial and anti-cancer properties.

XRD, SEM, and TEM analyses of synthesized nanocomposites revealed unique and specific patterns of nanocomposites [49]. The steric interaction among the CuO-TiO_2_-chitosan-escin matrixes and the intermolecular hydrogen bonding among the matrixes results in the formation of the CuO-TiO_2_-chitosan-escin nanocomposites. The average crystallite size of the CuO-TiO_2_-chitosan-escin matrix was 45 nm according to Debye-Scherrer’s formula. A series of Gram positive and negative bacteria, including *S. aureus* and *S. pneumonia*, were used to test the anti-microbial efficacy of CuO-TiO_2_-chitosan-escin nanocomposite materials. We found that the synthesized nanocomposites revealed substantial antimicrobial property using Gram-positive and Gram-negative pathogenic strains. Because of the ability of CuO-TiO_2_-chitosan-escin nanocomposites to induce ROS upon exposure to bacteria, their ability to impede bacterial growth mechanisms could be attributed to their ability to penetrate bacterial cell membranes and disrupt their growth mechanism [50]. Our results agreed with previous reports demonstrating that metal oxide nanoparticles could inhibit bacterial growth via ROS or membrane penetration.

Furthermore, in vitro tests of the anti-cancer efficacy of CuO-TiO_2_-chitosan-escin nanocomposites were performed using MOLT-4 blood cancer cell lines. Our initial cell viability assay determined the IC_50_ value ranging from 7 to 14 µg/mL within 24 to 72 h. The MOLT-4 cell line was analyzed following the initial measurement, and significant alterations in normal cell architecture were observed. Because of the presence of CuO-TiO_2_-chitosan-escin nanocomposites in the culture medium, the observed morphological changes could result from elevated ROS levels. Our results concurred with previous studies, demonstrating that chitosan nanoparticles could induce ROS, which ultimately contributed to the death of cancer cells [51,52].

Moreover, we executed acridine orange/propidium iodide (AO/PI) staining on MOLT4 cells treated with CuO-TiO_2_-chitosan-escin nanocomposites to determine whether these nanocomposites mediated apoptosis. A significant staining pattern for AO/EtBr was observed in the MOLT4 blood cancer cell line, indicating that the membrane integrity has been compromised. Inhibition of morphological changes by CuO-TiO_2_-chitosan-escin nanocomposites could be accredited to either the direct pathological impacts of ROS and oxidative stress caused by the nanocomposites, as previously described, or their ability to directly penetrate the cell membranes of MOLT4 cell lines and induced cellular stress.

To confirm the ability of CuO-TiO_2_-chitosan-escin nanocomposites to cause ROS in a cell culture medium, we conducted a ROS analysis experiment with the fluorescent dye DCFH-DA. Our results revealed that nanocomposites significantly increased the production of ROS in MOLT4 cells, indicating their potential to cause cytotoxicity, morphological alterations, and oxidative stress, as demonstrated in our preliminary studies [53]. Our findings were consistent with a report highlighting the ability of tailored nanoparticles to generate ROS in biological systems.

Finally, to confirm the stimulation of apoptosis events in the nanocomposites administered with MOLT4 cells, we identified CuO-TiO_2_-chitosan-escin nanocomposites by their ability to produce ROS, oxidative stress, and by penetrating the mitochondrial membranes of MOLT4 cells, which could lead to an increase in caspase activation. MDA is a primary products of lipid peroxidation; thus, its level represented the rate and intensity of lipid peroxidation in the cells. Compared with the control cells, CuO-TiO_2_-chitosan-escin nanocomposites (7 and 14 µg/mL) considerably increased MDA levels (nmol/gm) and significantly decreased GSH, SOD, and GR activities (*p* < 0.05).

A decrease in mitochondrial membrane potential levels is indicated by decreased dye accumulation in the mitochondria [54], which can be due to the excessive production of ROS. ROS could induce apoptosis by depolarizing the mitochondrial membrane, resulting in a rise in (lipid peroxidation) LPO by-products (TBARS) and a decrease in anti-oxidant enzyme activity (SOD and GR) [55]. These effects could be attributed by the induced ROS generation of CuO-TiO_2_-chitosan-escin nanocomposites. The outcomes of this study were consistent with previous research on colon cancer cells and Hela cells [56,57]. The activities of SOD, GR, GSH, and MDA were investigated to determine the levels of anti-oxidant enzyme status on MOLT-4 cell lines.

CuO-TiO_2_-chitosan-escin nanocomposites were tested for anti-cancer and anti-oxidant properties in MOLT-4 cell lines through endogenous anti-oxidant levels. Carcinogenesis produces a high concentration of ROS, which may contribute to oxidative damage [58]. Therefore, anti-oxidant enzyme levels may increase or decrease. In this study, SOD and GR activities were greater in untreated MOLT-4 cells (control) than in CuO-TiO_2_-chitosan-escin nanocomposite-treated MOLT-4 cell lines. Studies have revealed higher SOD and GR activities in various cancer cell lines [10,59]. Compared with the control cells, GSH content was lower, and TBARS content was higher in CuO-TiO_2_-chitosan-escin nanocomposite-treated cell lines. Low GSH levels were found in individuals with chronic alcoholic liver disease and liver cancer [60]. Tumor cells showed low GSH levels, which could be attributable to alterations in their defense system [56].

According to the present study, the anti-cancer activity of CuO-TiO_2_-chitosan-escin nanocomposites affected the state of anti-oxidant enzymes in MOLT-4 cells. The elevated activities of SOD and GR were dramatically lowered after treatment with CuO-TiO_2_-chitosan-escin nanocomposites. CuO-TiO_2_-chitosan-escin nanocomposites might have helped to eliminate superoxide radicals and H_2_O_2_ accumulation in MOLT-4 cell lines by acting as anti-oxidants such as SOD and GR. Based on our findings, CuO-TiO_2_-chitosan-escin nanocomposites mediate strong anti-bacterial and anti-cancer effects by generating ROS, oxidative stress, and inducing apoptosis. This effect could be due to the ability of the nanocomposites to bind to cell membranes, infiltrate cells, and cause endogenous stress [61]. Learning more about the molecular processes through which CuO-TiO_2_-chitosan-escin nanocomposites mediate anti-cancer effects in future studies would be advantageous for cancer therapy.

Apoptosis is thought to be triggered by various factors, including a change in the internal mitochondrial route that triggers the caspase cascade [62]. Caspase-8, which cleaves and activates effector caspases, regulates the activation of the extrinsic pathway. Cytochrome C is released during the intrinsic process, interacting with Apaf-1 to activate caspase-9, triggering PARP cleavage and apoptosis [63]. The morphology of the nucleus after 24 h of treatment with CuO-TiO_2_-chitosan-escin nanocomposites and Annexin V-FITC/PI staining revealed distinct dose-dependent apoptosis induction.

The present study revealed that the intrinsic mitochondrial pathway activates effector caspases, such as caspase-3, by hooking up intrinsic receptors. A damaged mitochondrial membrane triggers the caspase-9 cascade, triggering the apoptotic cascade via the mitochondrial pathway. As we observed, CuO-TiO_2_-chitosan-escin nanocomposites significantly stimulated caspase-3, -8 -9, and PARP cleavage, all required for cell apoptosis. Furthermore, certain caspase inhibitors prevented cell death caused by nanocomposites in MOLT-4 cancer cells. These nanocomposites induced caspase-9 activation, which promoted caspase-3 apoptosis, suggesting that CuO-TiO_2_-chitosan-escin nanocomposites played a role in mitochondrial apoptosis. Furthermore, the anti-oxidant significantly reduced the cytotoxicity of CuO-TiO_2_-chitosan-escin nanocomposites. Therefore, CuO-TiO_2_-chitosan-escin nanocomposites are believed to cause death in MOLT-4 cancer cells via superoxide production via the intrinsic mitochondrial pathway.

## 5. Conclusions

This study investigated whether CuO-TiO_2_-chitosan-escin nanocomposites can be used as anti-cancer therapies. Several characterization tests, including UV-vis, FTIR, and TEM analysis, revealed that CuO-TiO_2_-chitosan-escin nanocomposites could be synthesized in an eco-friendly manner. Based on in vitro experiments, CuO-TiO_2_-chitosan-escin nanocomposites were found toxic for MOLT-4 cells. At concentrations between 7 and 14 µg/mL, acute lymphoblastic leukemia cells (MOLT-4) exhibited dose-dependent cytotoxicity. The levels of GR, SOD, GSH, and MDA, which are sensitive to the mitochondrial pathways that control apoptosis were measured in MOLT-4 cells following the exposure to CuO-TiO_2_-chitosan-escin nanocomposites. CuO-TiO_2_-chitosan-escin nanocomposites significantly increased the expression of pro-apoptotic caspase-3, -8, and -9, while caspase-8 expression was significantly decreased. CuO-TiO_2_-chitosan-escin nanocomposites inhibited the proliferation of cancer cells. Using CuO-TiO_2_-chitosan-escin nanocomposites in cancer treatment could lead to the development of nontoxic and non-chemotherapeutic therapies. CuO-TiO_2_-chitosan-escin nanocomposites should be examined in greater depth in animal experiments to investigate their effect in in vivo models or cancer cell lines.

## Data Availability

The data that support the findings of this study are available from the corresponding writer upon reasonable demand.
